# Understanding the Capability of an Ecosystem Nature-Restoration in Coal Mined Area

**DOI:** 10.1038/s41598-019-55935-9

**Published:** 2019-12-23

**Authors:** Xiaoqin Cui, Suping Peng, Laurence R. Lines, Guowei Zhu, Zhenqi Hu, Fan Cui

**Affiliations:** 1 State Key Laboratory of Coal Resources and Safe Mining, China University of Mining and Technology (Beijing), Beijing, China; 2 Department of Geoscience, University of Calgary, Calgary, Canada; 3 College of Geoscience and Surveying Engineering, China University of Mining and Technology (Beijing), Beijing, China

**Keywords:** Ecological networks, Environmental impact

## Abstract

Ecosystem issues have been severely concerned and studied when the coal resource is one of major energy generators, and green mining innovation techniques involving artificial-restorations have addressed and significantly lessened negative impacts on the ecological environment. The ecosystem of a coal-mined area, however, is able to naturally restore with the processes of natural succession, similar to the human body system that has the immune ability to self-heal a wound over time if the wound does not deeply hurt the health. Here we analyze multiple discipline real data from two mining sites, and evidently show an ability of nature that the coal mining related problems such as geological cracks, damaged aquifers and destroyed soils in Quaternary period can naturally recover around a half-year after the end of mining. Our results temporally and spatially demonstrate that the damaged ecosystem has a capability of unaided nature-remediation from the ground to the subsurface, which is very useful to the countries worldwide with abundant coal reserves and intense energy demands for their development.

## Introduction

**Figure 1:**
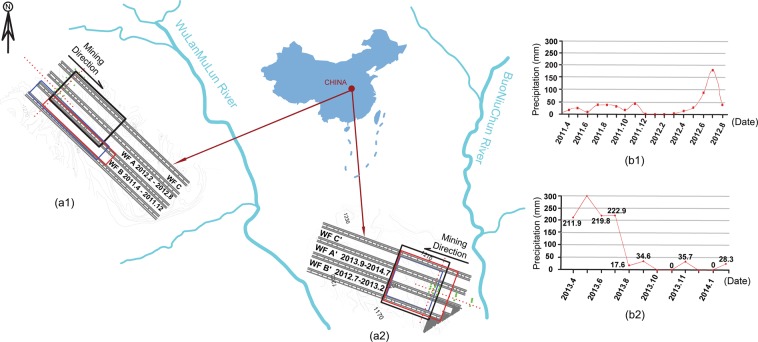
Location and map of the mining sites and corresponding local precipitation records. (**a**) Locations of the workfaces (WFs), time-lapse seismic survey (the black solid lines), electrical prospecting survey (the red solid lines) and ground penetrating radar survey (the blue solid lines), as well as the observation data (the red dots) and the soil sampling points (the green dots) in the BLT (the red dots:45 × 45, the green dots:12) site (a1) and the DLT (the red dots: 51 × 34, the green dots:20) site (a2). It annotates that the sites of the BLT and the DLT have different mining directions and different mining date. (**b**) Diagrams of the precipitation records. Note that rich moisture naturally compensates at end of mining in the BLT site (b1), while massive precipitation as water source alters at beginning of mining in the DLT site (b2).

Comprehensive ecological nature-restoration investigations on the crack structures, the aquifers and the soil qualities over the coalbed mining areas are essential to assist a program to control any potential environmental problems including excessive artificial repairing^1–8^. A coalfield ShenDong (SD) located in western China, a rapid and efficient coalbed mining industry base, is the third largest coalfield in the world. Annual production grew to 33 million tons from only four the longwall workfaces (over 300 × 4000 m) in 2017, which is about 48% of China’s total production. The longwall workface (WF) mining is an underground coal extraction method based on a systematic removal the coalbeds in a series of workfaces (WFs) that move through the coal deposit, and it commonly occurs as multiple WFs in parallel^9–11^. In the SD area, there are two main rivers, WuLanMuLun and BuoNiuChuan, and other creeks (Fig. 1a), the main recharge of the ground and the underground water sources is precipitation only with 357 mm per year. The annual average evaporation, however, is around 2457 mm, which makes the water resources inadequate. The aquifer is comprised of pore phreatic Cenozoic loose strata, fissure diving Mesozoic clastic rocks, confined water, and phreatic of pore, and fissures of pyrogenic rocks, so that the ground surfaces suffer from desertification and potential desertification. Due to the short term rainstorm erosion and long term wind erosion, accounting for 77% and 96% of total SD area respectively, both types of erosion occur alternately in time and space, which also makes soil loss very serious - resulting as coarse texture, poor structure, low fertility, and poor soil conditions with corrosion and impact resistance in the area. The coverage of restoration in the aerial seeding area is over 65%, and the forest and grass compose more than 59.4% of the area now. In other words, natural geography, hydrogeology and vegetation situations establish ecological environment fragility in the SD area^12,13^. Geologically, the coalfield SD is covered by loose Quaternary sediments at a shallow zone of around 40–65 m thickness and the main coalbed is deposited at 150–200 m depth with 5–7 m thick belonging to Middle and Lower Jurassic. Two mining sites 25 km away in the SD coalfield, BuLianTa (BLT) and DaLiuTa (DLT), have been investigated for an issue of ecological environment relating to dynamic coalbed mining. Figure 1a presents a map of the mining WFs related to our research. This area contains a 3D seismic survey to determine seismic reflectivity for the earth’s crustal structure, an electrical resistivity (ER) survey to predict resistivity for the rate of water content and a ground penetrating radar survey (GPR) to probe dialectic for the subsurface characterizations. Also, the locations of the observation data (the red circles), the soil sampling points (the green squares) and topographic information are marked on the map. In Fig. 1a, the WF A in the BLT and the WF A′ in the DLT are research targets and have undergone pre-mining, mining and post-mining stages. The start of the mining was from February 2012 and September 2013, respectively with the end of mining being in August 2012 and July 2014, respectively. Corresponding adjacent WF B and WF B′ have completed mining production from April to December in 2011 and July 2012 to March in 2013, respectively, while WF C and WF C′ have not been considered a mining work yet. Thus, the WFs C and C′, the WFs B and B′ are defined as the pre-mining stage and the post-mining stage respect to the mining stage of the WFs A and A′. Following the certain mining progressing of the WFs, the time-lapse acquisitions of seismic exploration, ER and GPR have been sequentially exerted on the pre-mining, the mining and the post-mining stages with 10 days for each stage^14–19^. Figure 1b presents the precipitation records for the BLT and the DLT sites in the mining production period which is an important variant and factor to the ecosystem. In this paper, convincing results analysis would support the observations that changes of the damaged ecological environment are in a natural renewal trend after the mining stage^4,7,20–22^. We conclude by identifying natural restoration knowledge gaps from our understanding and summarize the case study in terms of the nature-rehabilitations for the coalbed production neds.

In this study, we newly used the time-lapse method to acquire and observe, process and interpret multiple discipline 3D geo-data at two sites of the coalbed mining areas to evaluate ecosystem changes. The time-lapse schedule matched the pre-mining, mining and post-mining stages, the multiple discipline 3D data focused on the shallow, middle and deep different depth. Therefore, we temporally and spatially analyzed ecological parameters changes from the lithosphere, hydrosphere and pedosphere to innovatively illustrate that coalbed mined areas had the capability of an ecosystem nature-restoration.

## Results

### Structures

**Figure 2:**
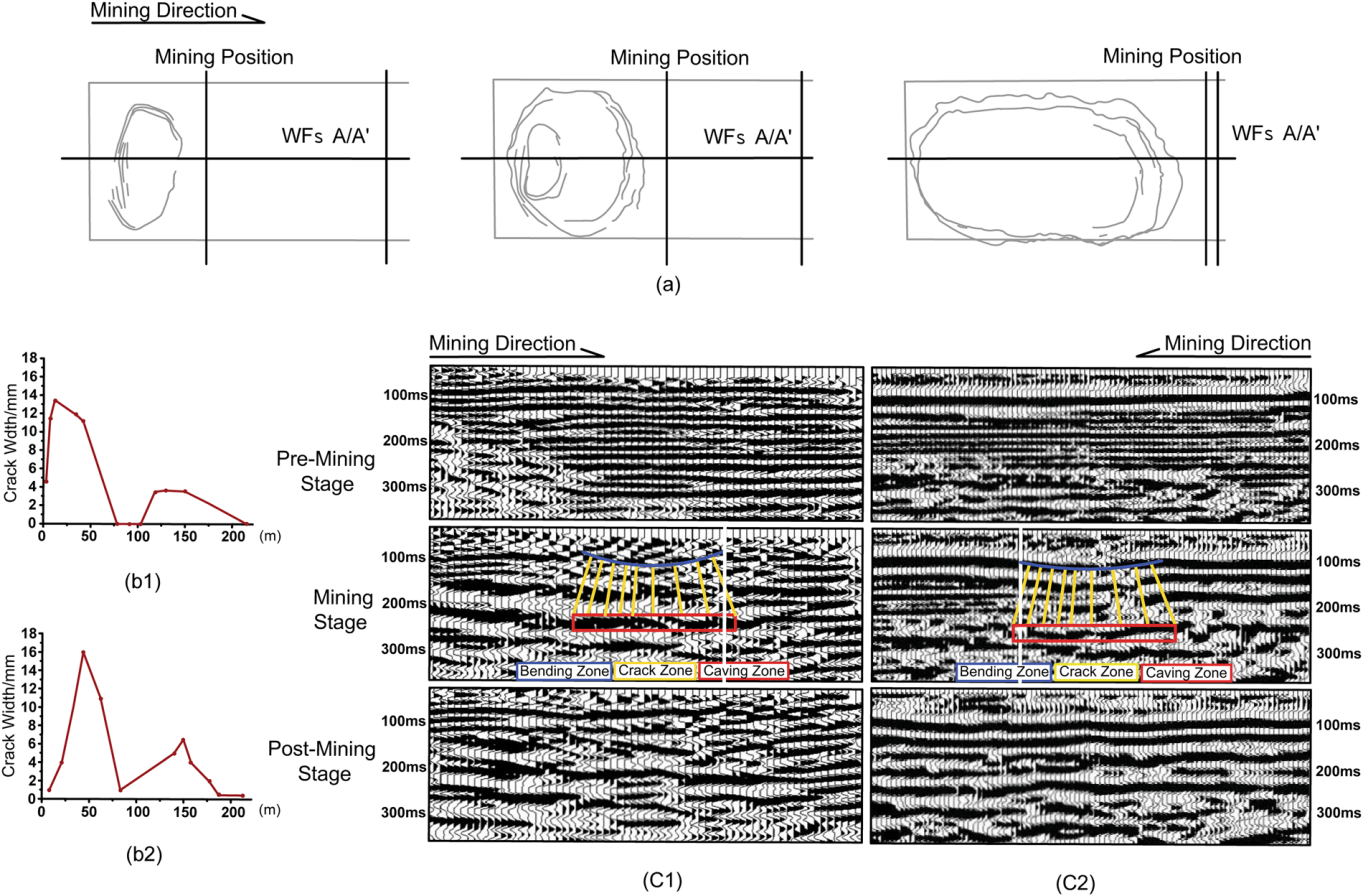
Demonstration of geological structures variation with mining actions. (**a**) Sketch of surface crack developed shapes and observation point locations (the dotted points). Note that the shapes of both BLT and DLT have the same regulation which statistically analyzes several measurements from different observation points at different mining positions (The dashed lines). (**b**) Trends of dynamic crack width function to the distance between the crack location to the mining position on the ground. The results count 5 cracks for the BLT site (b1) and 3 cracks for the DLT site (b2) with 20 days observation for each. (**c**) Time-lapse seismic inline time sections that is parallel to the mining direction for the BLT site (c1) and the DLT site (c2). Note that three vertical destroyed zones occurred behind the mining position in the mining stage for both BLT and DLT sites (The bending zone is interpreted with blue color; the yellow stick represents the crack; the red square indicates the caving zone). The white dashed lines denote the mining position.

When the partial coalbed is mined in the WFs, the equilibrium states of the stress in the relevant rock mass are broken that the stresses have to redistribute achieving a new balance, in which the pressure bearing zone and the decompression zone are formed in the top and bottom of the slates^23^. Once the effect of deformation spreads to the ground surface, the cracks would be generated on the ground surface that include dynamic cracks opening temporarily and edge cracks remaining permanently open. Figure 2a shows the cracks from beginning with dynamic one to the end with the permanent one in the entire mining production stages that the cracks experience with ellipsoid, circle and then rectangle patterns, especially in situations in which some of dynamic cracks automatically disappear in the post-mining stage on the ground subsidence area. Meanwhile, the crack distributions imprints deviate into the adjacent post-mining stage WFs B/B′ which promotes the cracks occurrence and distribution for the mining stage WFs A/A′. Referring to the stress measurements in the 3 m depth, many crack aperture observation data from different locations statistically prove that the ground surface dynamic cracks variation trends can be roughly divided into four steps: initial development, initial closure, secondary development and secondary closure (Fig. 2b). Even though the secondary closure time span is slightly longer than the initial closure there is relatively intense activity and concentration of the stress distribution, and it demonstrates that the ground surface dynamic crack life ranging is around 20 days from the initial opening to the final natural repairing closure.

At the underground, as a result of the pressure relief, the roof rock mass of the WFs moves down and deforms to form three vertical zones: caving zone, crack zone and bending zone from the coalbed roof to the ground^24^ (Figs. 2C1 and 2C2) and a uniform subsidence area on the ground^25^. The maximum subsidence ratio of the ground surface both are around 2% of the coalbed thickness in the DLT and the BLT sites by using Global Positioning System (GPS) observations. Comparison the time-lapse seismic processed data between the pre-miming stage data (The baseline survey) and the mining and the post-mining data (The monitor survey) exhibits that the structures of the mined zone and three vertical deformed zones present incoherent reflections and lower frequency in the mining stage due to the destroyed rock mass that attenuates and scatters the seismic wavefield with the different phases. In the post-mining stage, the structures have been compacted reference to the mining stage that illustrate the reflections having more coherency and higher frequency contents, while the earth stratum are few reconstruction to the original lithosphere even though the timing past one and half years after the end day of the mining (Fig. 2c)^14,16,17,19^.

In general, the compacted masses are from the dropped overburden layers, the height of the destroyed zone formed by weak rock overburden is 9–12 times of mining thickness, 12–18 times of mining thickness of medium-hard rock overburden, 18–28 times of mining thickness of hard rock overburden. The heights of the caving zone and the crack zone account for a quarter and three of four of the total destroyed zones, respectively. The surface would connect to the mined zone through the mining cracks once the surface collapses, therefore, the coalbed mining production affects not only the subsurface the rock structure but also the upper primary aquifer structure that changes groundwater runoff and discharge conditions.

### Aquifers

**Figure 3:**
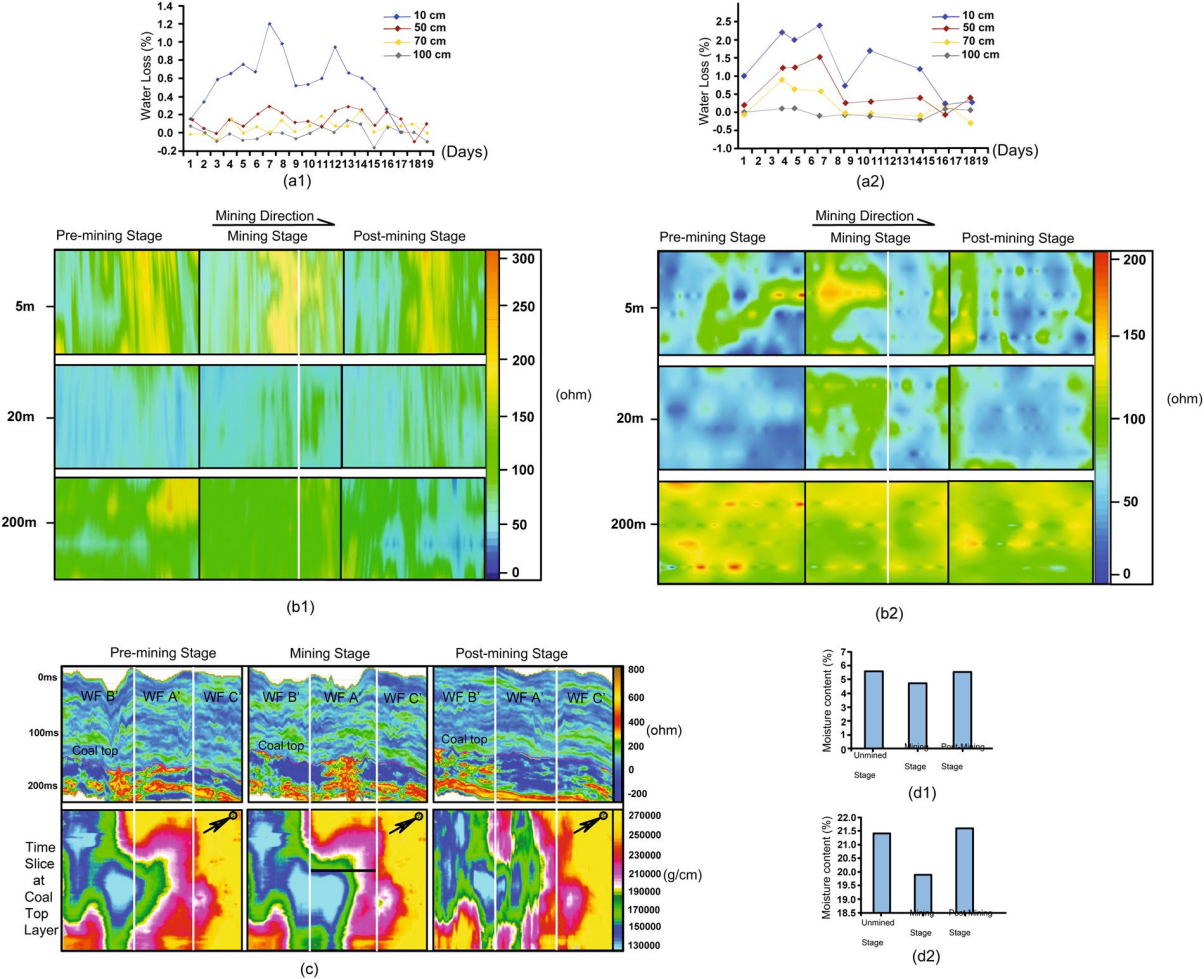
Demonstration of the aquifer changes with the time-lapse data at the ground and the underground. (**a**) The charts of soil water loss for the BLT site (a1) and the DLT site (a2). The curves depend on the distance between observation points to different cracks as 10 m (the blue), 20 m (the red), 70 m (the yellow) and 100 m (the grey) that used TDR300 moisture content speedometer and GPS instruments to measure water content loss at 5 observation points with 11 times of repeating measurements for 20 days. (**b**) Visualizations of the apparent resistivity at different depths (5 m, 20 m and 200 m) for the BLT site (b1) and the DLT site (b2). It results of the time-lapse electoral prospecting inlines making the depth slice. The inline direction that is parallel to the mining direction that is marked on the mining stage. The white dashed line defines mining position. (**c**) Image of inversed resistivity of time-lapse seismic crossline time section (The top) that is perpendicular to the mining direction and time-lapse time slice at coal top layer (The bottom). The white dashed lines indicate workface boundaries and the dark dashed line presents the mining position. (**d**) Chart of the average water content ratio. GPR is used to measure four times for the sandstone layer (d1) and clay layer (d2) at the central line of the WF A′ in the DLT site.

The hydrology is one of the main concerns regarding ecological environment problems, in which the mining cracks lose and redirect the water into the mining WFs. Thus, to further analyze and study the original distribution of the aquifers at the pre-mining stage and the changes of aquifers in the ground and the underground at the mining and the post-mining stages that can provide the scientific utilization basis for the water resources remediation issues^2,14,26–32^. With the large-scale and high-intensity subsurface mining, it will inevitably deform and destroy the overburden layers of the coalbed, which increase the layer permeability of the subsurface and drastic changes in the recharge, runoff and drainage of the water resources system meaning the surface water, diving water and partially confined water flow into the mined void zone through the cracks, leading the mined void zone to be the channel and storage space of water diversion. Meanwhile, the porosity and the number of pore sizes of soil capillary holes around the cracks increase significantly with the extension of cracks, which results in the aquifer seepage, the increase of evaporation in the contact area between the water and external air, and even the accelerating of rainfall moisture penetration into the deeper ground. Several ground dynamic cracks generated at very closer dates but different locations on the WFs A and A’ are used to examine the loss of average water content. The results are shown in Fig. 3a. The lost water content of the ground grows with the increase of the distance in the range of less than 70 cm from the crack position, which means the mining production formed cracks mainly affect the water contents in the vicinity zones. However, on the other hand, the crack developments are gradually closed about 20 days, the surface water infiltration and evaporation degree of each section tend to be in accordance, and the influence moisture action around the cracks has naturally ended, the water content has no loss thereafter.

It is believed that different rocks have different resistivity, which is related to the mineral composition, water cut, porosity, temperature, and pressure^33^. At the subsurface, the more cracks or mass failure formed in the rock, the larger the porosity of rock would be. The more water contents in the rock, the better the conductivity and the smaller apparent resistivity, vice versa^18^. Figure 3b presents the depth slices of ER results of the apparent resistivity investigations at average depths of 5 m, 20 m and 200 m in which the apparent resistivity in the shallow zone (5 m –20 m) increases from the pre-mining stage to the mining stage, then decreases back to pre-mining status almost at the post-mining stage, the apparent resistivity in the deeper zone (200 m) decreases from the pre-mining stage to the mining stage and partly gets back to original value at the post-mining stage. Because the apparent resistivity is reciprocal to the water contents, it can be explained that the shallow water at starting stage of the mining production drains through the three vertical destroyed zones into the mined void zone which makes the shallow and deeper apparent resistivities increase (5 m and 20 m), whereas the deeper layers (200 m) has a less water with higher apparent resistivities values in the pre-mining stage, but they decrease in the mining stage that illustrate the water of overburden layers dropped down to the mined zone. After a half year of the post-mining stage, the shallow water source system automatically redistributes to an avail level which follows the destroyed zones self-repairing according to the processes of natural succession, so that the apparent resistivity returns to the pre-mining stage values, while the deeper coalbed mined zone (200 m) restore massive water from overburden resulting of the lower apparent resistivity value except partial back to the pre-mining stage with higher apparent resistivity value, it means that the mined void could become an artificial subsurface reservoir that is extremely useful to the ecosystem after the water recycle treatment^34,35^.

Figure 3c provides seismic inversed resistivity crossline time sections (The top) and inversed impedance time slices that equal to the rock velocity multiples density (The bottom). There is no doubt that the “footprint” of the mining production is visualized on the seismic inversed data^14,16,17,19^. In the time-slice image for the production zone, the range of the lower impedance in the mining stage WF A’ is larger than the pre-mining stage WF A’ (The blue color), but smaller than the post-stage WFA’ respect to the mining production position; it denotes that the mining action destroys the rock structure to decrease the velocity and density as well as increase void space so that the mined space possibly develops to an impounding reservoir. According to the inversed resistivity crossline time section, the water contents above coal top layer have rehabilitated at the post-mining stage, although the aquifer recovery rate is much affected by the climate which is one of the nature succession processes^36^ (See Fig. 1b). The water content in the deeper coalbed mined zones partly repairs back to the pre-mining stage, the rest zones accumulate the water to make the rock resistivity decrease that all agree with ER results in Fig. 3b.

The result of the time-lapse GPR for WF A′ in Fig. 3d represents that the average moisture contents ratio of sandstone (0–8 m) and clay (0–9 m) in the mining production stage are the smallest, which are 4.89% and 19.58%, respectively, while the ratio in the post-mining stage are basically the same as the unmined stage^15^. This GPR conclusion agrees with the electrical prospecting and seismic exploration results from the water loss that occur in near surface soil loosening caused by the mining effect. This water loss could terminate when the destroyed structure zone stops development and the drainage path renews. Therefore, the ability of the auto-remediation of the water content follows the regulation of the destroyed structure restoration, in which water content in the shallow zone seriously influences the soil properties.

### Soil

**Figure 4:**
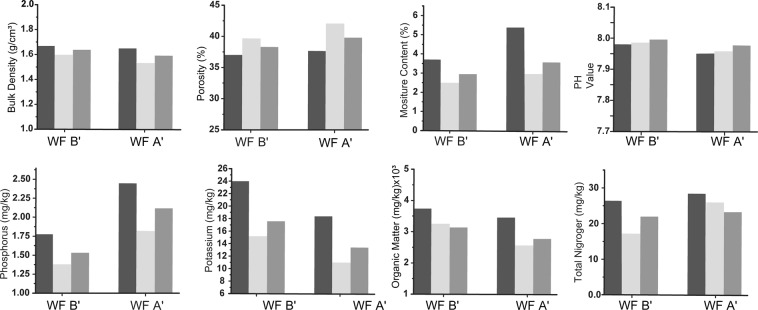
The soil physical and chemical indications for the DLT site. The left and right parts of each chart illustrate soil properties (at the vertical axis) in the post-mining stage (WF B′) and the mining stage (WF A′) (at the horizontal axis), respectively, in which the colors of the black, light gray and gray represent the unmined, the mining cracked and post-mining stages, respectively, and each sampling strictly accordance with requirement method and scientifically take measurements.

The soil is a complex natural body with its own process of occurrence and development, unique morphological and fabric characteristics in whole ecosystem^6,7,17,37–40^. The ground and the underground movement and deformation caused by coalbed mining will weaken and change physical, chemical and biological soil indications. The bulk density, the porosity and the moisture content are basic physical indications with a great influence on the soil permeability, infiltration performance, water holding capacity, solute migration characteristics and erosion resistance. Indeed, those physical properties are a comprehensive reflection of the internal quality, and the abilities of fixation and release of nutrient elements of the soil. In addition, the organic matter, pH value, and phosphorus, potassium, nitrogen elements are important factors affecting nutrients and the exchange of ions, movement, transformation and the survival of microorganisms in the soil that represent fertility and plant growth characters. Figure 4 has a point of view from a single WF A′ or B′ in DLT site, the soil physical and chemical indications all respond to the mining stages, the influence degree of the damaged zones is that the indications in the cracked mining stage zone and post-mining stage zone are weaker than those in the unmined stage zone. According to the comparison with WFs A′ and B′, the physical and chemical properties in the earlier WF B′ are better than those of later WF A′. It manifests that coalbed mining zone causes certain disturbance to soil biological qualities, but it has a tendency to natural restoration rapidly with the passage of time and gradually remediates to closer to the unmined zones. In fact, some woody species or vegetation can fix and accumulate nutrients in sufficient quantities to provide the soil self-sustaining quickly.

## Discussions

The capability of an ecosystem natural restoration in the coalbed mined area not only relates to the mining design, but also connects the nature interactions of regional climate, biosphere and geographical environment^41–47^, whereas these natural factors are often neglected. The mining sites of the DLT and the BLT are located in the aeolian sand area and belong to longwall WFs that increase the area of uniform subsidence and reduce the damage degree of the overburden layers so that benefit to use the self-repairing capabilities to the ecosystem^41,48,49^. In the DLT and the BLT mining stages, the mechanical mining force produce the coals as well as cut original continuous homogeneous layers into the rock blocks with different sizes and generate geological discontinuity body^33^. The mining action fundamentally deforms the strength property of the rock mass and the stress balance in the underground, result in the formation of the caving zone, the crack zone and the bending zone from the coalbed roof to the ground^24^ (Fig. 2C), even the deformation upwardly transmitted to form dynamical crack geological structures at the alluvium period layers, which had signed as the surface cracks in the subsidence area on the ground. In view of the groundwater with terms of tectonic stress, no matter the mining area is in the compressive or the tensile fields, the coalbed mining destroys a stable assemblage to the aquifer, the confining bed and the aquiclude zones, in which the groundwater in the overburden strata of the coalbed flow downward to the mined zone along the crack zone and store there. Moreover, the dynamical cracks on the subsidence area of the ground increase the external contact area of the soil with the air that accelerates the evaporation of the groundwater. In term of the soil under the mining procedures, due to factor that the ground surface connection has been cut off by existed dynamical cracks, the soil character sign as the loosed and the lost, the water retention capacity weakened and the nutrients diminished. Meanwhile, the soil chemical composition, the microbial population structure of the plant rhizosphere and the soil activity enzyme have changed also. In a word, the dynamical crack generating, groundwater level and the soil nutrient degradation narrated above illustrate that ecological environment have been destroyed by violent mining processing. However, the deformations are dynamic, because of the coalbed mining step continues forward, i.e., the mechanical mining force makes the position of the dynamic cracks to move forward with the coalbed mining direction, the dynamic cracks automatically self-repair to close following the alternative tectonic stresses of the compressive and the tensile in the subsurface. With the stress measurements investigation, we find that the period of the dynamic cracks self-repairing is around 20 days with four steps as initial development, initial closure, secondary development and secondary closure (Fig. 2b). The procedure reestablishment of the damaged deep geosphere needs a diagenesis under certain compression, temperature conditions and geological time. In the case of the groundwater, as the tectonic stress conditions change caused by mechanical mining over again, the groundwater system controlled by geological tectonic stress will produce corresponding the changes to achieve a new equilibrium state^10,45^. The closed dynamic cracks and the compressed lithosphere effectively prevent the aquifer depletion. We study that the groundwater content is accordance with the dynamic cracks recovery in the time and space to attain the aquifers restoration. Furthermore, the rainfall recharging raises the groundwater level as well. To the soil, it has an endogenous ability of the biodegradation and the migration that undergoes through physical, chemical and biological reactions. By reason of the existed initial dynamic cracks, the nutrient elements in the air easily entered the soil, and the available states of the elements in the soil moved up slowly with the water evaporation, as well as the porosity, the permeability and the structure of the soil improved at the same time^37,46^. On the other hand, under actions of the time lapse, the rainfall recharging and the gravity, the loosed soil gradually compacted and tended to be stable that makes the bulk density, the water retention capacity and the nutrient contents of the soil increasing at certain extent. In addition, the elements in the gangues, the coal ashes and some wastes can exchange with the soil elements under rainfall leaching action which enriched the availability elements for the soil and improved the soil structure^43,49^. Especially, some of the microbes from initial destructed plant litter, saprophyte, exudates and decayed roots, as well as the faeces and the exudates of the animals circularly exchange with the pH value, the phosphorus, the potassium, the nitrogen elements and the organic matter in the soil so that significantly reclaim the damaged soil. Therefore, the natural abilities of the rain, the wind, the gravity, the sunlight, the plant and the natural seed bank plus the mechanical mining force achieve the dynamical geological structure recovery, groundwater lever restoration and the soil nutrient reclamation by undergoing the physical and chemical and biological functions after mining action. It manifests that the mined areas have strongly a capability of natural restoration for the ecosystem.

Researched on the geological structures of the surface and subsurface, the water contents and soil nutrient elements from the pre-mining, mining and post-mining stages, it notes that in the aeolian sand mined area, the dynamic fractures in the near surface can be self-remediated in the 20 days, the water content in the subsurface will automatically restore within a half year and the nature rehabilitation on soil nutrient is around one year. Therefore, with similar geological conditions and mining technology, all the mined areas have a specific capability of an ecosystem nature-restoration.

## Conclusions

The idea is that the mined area in the coalfield can be restored – and will quickly restore itself – to build a new balance for the ecosystem without external aids. From a new perspective, the promoted scientific understanding of a relationship between the mined zone and the ecosystem is the best of ecological engineering that nature-remediation can be achieved at low cost and self-sustaining in the long term. In order to avoid unreasonable anthropogenic interference leading a further degradation of ecosystems, therefore, we stress that the post-mining sites have strong ability to recovery spontaneously and some technical reclamations should take such natural restoration processes into consideration.

## Methods

### Seismology

Exploration seismology is the most widely used geophysical method. Seismic waves produced by a controlled source propagate through the subsurface structure, carry on the reflected characteristics of the rock and then recorded in digital on the surface^14,16,17^.

1 $$S(t)=W(t)\ast R(t).$$

Where *S*(*t*) is a seismic data recorded in time on the surface. *W*(*t*) denotes the source wavelet. *R*(*t*) indicates the reflectivity of the subsurface rock and the start * expresses convolution. In general, the digital seismic data records need to do signal processing through some of mathematic algorithms. For example, the deconvolution is for increasing temporal resolution by compressing the seismic wavelet; the velocity analysis tends to correct the hyperbolic curvature into flat reflected events respect to the normal incident traveltime; the stack is for enhancement the signal-to-noise ratio by reducing seismic data volume to the zero offset seismic section; the migration performs for increasing lateral resolution by collapsing diffractions and moving dipping events to their supposedly true subsurface positions. After seismic data processing, an inversion algorithm can transform the processed true seismic amplitude into a quantitative rock-property description of the subsurface structure:

2 $${\rm{G}}x=d.$$

Where G is a linear operator depending on the geometry. *x* is the column vector for the unknown parameters description of the rock properties. *d* is the column vector for the migrated seismic data.

A time lapse data is obtained by a seismic survey repeated acquisition after a period of time. Time lapse surveys are acquired in order to observe target changes during production that may not be detectable in conventional seismic method.

### Electrical resistivity (ER)

Electrical resistivity prospecting is an electric profile method for imaging subsurface structures from electrical resistivity measurements made on the surface. The precondition of electrical resistivity prospecting is the difference of electrical conductivity among underground rocks and minerals. Its principle is the same as that of the ordinary resistivity method as^14,18^.

3 $$\rho =K\frac{\Delta U}{I}.$$

Where *ρ* is resistivity. *K* indicates coefficient of the devices. Δ*U* denotes the potential difference and *I* is current. By observing and studying the distribution of the current field in the survey, the questions of the hydrological, environmental and engineering can be answered.

### Ground penetrating radar (GPR)

GPR uses electromagnetic radiation to detect the reflected signals from the changes of the material properties, the voids and the cracks in the subsurface. The principle of GPR is similar to the seismology, except GPR methods implement electromagnetic energy rather than acoustic energy^14,15^,

4 $$V=\frac{C}{\sqrt{{\varepsilon }_{r}}}.$$

Where *V* is the electromagnetic wave velocity. *C* is the velocity of electromagnetic wave propagation in vacuum. *ε*_*r*_ denotes relative dielectric constant of underground medium. It notes that the waves may be reflected at the boundaries where the subsurface electrical properties changed rather than the subsurface mechanical properties are the case with seismic energy. GPR usually uses high-frequency radio waves in the range from 10 MHz to 2.6 GHz and apply in a variety of media, including rock, soil, ice, fresh water, pavements and structures.

### Soil bulk density (BD)

Soil bulk density (BD) is the solid particle weight per unit volume of the soil when the natural structure of the soil is not destroyed. The unit is g/cm^3^. Detailedly, the soil samples are acquired through repeating three times with ring knife device and immediately cover the two ends of the device in order to avoid evaporation. The sample immediately weigh (accurate to 0.01 grams) and record. Then the sample containing box was dried (accurate to 0.01 g) and the soil moisture content was measured.

5 $$BD=\frac{100G}{V(100+W)}.$$

Where BD is the soil bulk density. *G* is the weight of the wet sample. *V* is the volume of the soil sample. *W* is the water contents of the sample. Soil bulk density (BD) is determined by the mineral composition, soil texture, soil structure and tightness.

### Soil porosity (P)

Soil porosity is the percentage of the soil pore volume per unit volume of the soil in natural condition, and it is a quantitative index to measure the soil pore. It is

6 $$P=\frac{p}{V}.$$

Where *p* is the pore volume, while *V* is the soil volume. The soil porosity P directly affects the soil capacity of the water and heat exchange, the soil permeability, the water retention and the plant growth. Therefore, the soil porosity is not only an important attribute of the soil, but also an important indicator of the soil fertility.

### Soil water content (W)

Soil water content is the ratio of the soil moisture mass to dry soil mass.

7 $$W( \% )=\frac{{V}_{a}-{V}_{d}}{{V}_{d}}\times 100 \% .$$

Where *W* is the soil moisture. *V*_*a*_ is the soil sample quality before drying. *V*_*d*_ is the soil sample quality after being dried. The soil structure changes, the specific surface area increases, and the contact area between the surface water and external air is enlarged, which is an important stage of the surface water research.

### Soil pH value

Soil pH value was measured by PHS-3D pH meter, the soil sample was passed through 1 mm sieve, and the air-dried soil sample was placed in a beaker with 25 g of deionized water. The soil was fully dispersed by adding water 50 mL, and the soil was completely dispersed for 1 hour to make it clear. The ball bubble of pH glass electrode was inserted into the lower suspension liquid, gently swayed, the water film on the glass surface was removed, the electrode potential was balanced, and then the calomel electrode was inserted into the upper liquid, and the pH was measured by pressing the reading switch.

### Soil phosphorus (P)

Using pH8.5 NaHCO3 flow as digestion, the result of the extracted phosphorus has an excellent correlation with the phosphorus in the soil. Under certain acidity, the extracted phosphorus has been returned to the blue color by using molybdenum stadium anti reagent. The density of the blue color is proportional to the content of phosphorus in a certain concentration range that the content of phosphorus can be determined.

8 $${\rm{P}}({\rm{mg}}/{\rm{kg}})=({\rm{mg}}/{\rm{L}})\frac{50}{10}\times \,100/{\rm{m}}.$$

Where P is the soil phosphorus. mg/L denotes the number of phosphorus concentrations on the standard curve. The number 50 denotes the total volume of chromogenic solution. The number 100 indicates the total volume of pH 8.5 NaHCO_3_. The number 10 expresses total volume of the suction filtrate. m is quality of air-dried soil samples. The soil phosphorus is an element in the soil that can be divided into inorganic phosphorus and organic phosphorus. The content of phosphorus in the soil directly determines the current capacity of phosphorus supplement in the soil.

### Soil potassium (K)

Estimation potassium K in the soil uses the flame photometry method with natural 1molL-1NH4Ac solution. The principle is that NH4^+^ was exchanged with K^+^ of the surface of the soil colloid when the soil sample was in the solution. Therefore, the exchanged K^+^ with the K^+^ of the water would entirely be determinate. The formula for the calculation of the potassium in the soil is

9 $${\rm{K}}=\frac{{c}_{1}{V}_{1}}{{\rm{m}}}.$$

Where K is the extracted potassium in the soil. *c*_1_ is the value of the potassium concentration in the liquid that obtain from the checking standard curve. *V*_1_ is the volume of the extracting flow. m is a quality of the soil sample.

### Soil organic matter (SOM)

Under the condition of electric heating, the organic matter in the soil was oxidized by using a quantitative potassium dichromate sulfuric acid solution. The remaining potassium dichromate was titrated with the ferrous sulfate standard solution, and the silica as additive was used as the actual blank calibration. According to the difference of oxidant mass before and after oxidation, the content of soil organic matter is that the amount of calculated organic carbon multiple the coefficient of 1.724.

10 $${\rm{SOM}}=\frac{({V}_{0}-V){C}_{2}\times 0.003\times 1.724\times 100}{m} \% .$$

Where SOM is the organic substances in the soil. V_0_ is the volume of the standard ferrous sulfate solution consumed in blank titration. *V* is the volume of the ferrous sulfate standard solution consumed in determination of the sample. *C*_2_ is the concentration of the ferrous sulfate standard solution. 0.003 is number of the carbon atom of the Molar mass. 1.724 is a coefficient of the organic matte converted from the organic carbon. M is a quality of the drying sample.

### Soil nitrogen (N)

Before the sample was boiled, the nitrite nitrogen in the sample should be oxidized to nitrate nitrogen with potassium permanganate and then all nitrate nitrogen should be reduced to ammonium nitrogen by reducing iron powder. After boiling with concentrated sulfuric acid with the participation of accelerator, various nitrogen-containing of the organic compounds were transformed into ammonium nitrogen. With a complex high-temperature decomposition reaction, all nitrogen-containing of the organic compounds were transformed into ammonium nitrogen by boiling concentrated sulfuric acid with the participation of accelerator. The alkalized ammonia distilled is absorbed by using boric acid which titrated with acid standard solution. Finally, the total nitrogen content of the soil is obtained.

11 $${\rm{N}} \% =\frac{(V-{V}_{0})\times {C}_{H}\times 0.014}{m}\times 100.$$

where *V* is the volume of acid standard solution in the titration. *V*_0_ is the volume of acid standard solution used in the blank titration. *C*_*H*_ is the concentration of acid standard solution.

0.014 expresses milli-Molar quality of the nitrogen atom. m is quality of drying soil sample.

## Supplementary information

Dataset 2b1, Dataset 2b2, Dataset 3a1, Dataset 3a2,Dataset 3d1, Dataset 3d2, Dataset 4

## Data Availability

All datasets and results are available from the corresponding author on request.
