# Supplementary material for: Understanding the Capability of an Ecosystem Nature-Restoration in Coal Mined Area
Source: Sci Rep. 2019 Dec 23;9:19690. doi: 10.1038/s41598-019-55935-9 (PMC6928156; doi:10.1038/s41598-019-55935-9)
Supplement: Supplementary file 1 — Dataset 2b1, Dataset 2b2, Dataset 3a1, Dataset 3a2,Dataset 3d1, Dataset 3d2, Dataset 4 [file 41598_2019_55935_MOESM1_ESM.docx]

**Understanding the Capability of an Ecosystem Nature-Restoration in Coal Mined Area**

Xiaoqin Cui^1^; Suping Peng^1*^; Laurence R. Lines^2^; Guowei Zhu^3^; Zhenqi Hu^3^; Fan Cui^3^;

*^1^ State Key Laboratory of Coal Resources and Safe Mining, China University of Mining and Technology (Beijing), China*

*^2^ Department of Geoscience, University of Calgary, Canada*

*^3^ College of Geoscience and Surveying Engineering, China University of Mining and Technology (Beijing), China*

| Observe days | Distances from mining stage | Crack1 width(mm) | Crack2 width(mm) | Crack3 width(mm) | Crack4 width(mm) | Crack5 width(mm) |
| --- | --- | --- | --- | --- | --- | --- |
| 1 | -4 | 3.2 | 2 | 4.5 | 7.1 | 4.8 |
|  | 3 | 5.8 | 11.9 | 16.2 | 9.2 | 13.7 |
| 3 | 14 | 6.2 | 12 | 16.1 | 8.3 | 24.4 |
| 4 | 25.3 | 5.6 | 13.3 | 12.3 | 5.2 | 22.9 |
| 5 | 38.9 | 2 | 11.4 | 11.5 | 2 | 19.1 |
| 6 | 57 | 0 | 0 | A1 | 1 | 1 |
| 7 | 80 | 0 | 0 | 0 | 0 | 0 |
| 8 | 93.5 | 0 | 0 | 0 | 0 | 0 |
| 9 | 104.5 | 0 | 0 | 0 | 0 | 0 |
| 10 | 118.6 | 2 | 7.1 | 3.9 | 2 | 2 |
| 11 | 131 | 2 | 7.3 | 4.3 | 2 | 2 |
| 12 | 142 | 2 | 7 | 4.3 | 2.1 | 2.1 |
| 13 | 154 | 2 | 7 | 4.3 | 2.1 | 2.1 |
| 14 | 168.5 | 1.3 | 5 | 3 | 2 | 2 |
| 15 | 186 | 1 | 2 | 2 | 1.6 | 1.5 |
| 16 | 202 | 0.5 | 1 | 2 | 1 | 1 |
| 17 | 218 | 0 | 1 | 1 | 1 | 0.5 |
| 18 | 233 | 0 | 0 | 0 | 0 | 0 |
|  |  |  |  |  |  |  |

Supplementary data for Figure 2b1: Dynamic crack variation trend in the BLT site

Supplementary data for Figure 2b2: Dynamic crack variation trend in the DLT site

| Observe date | Distances from mining stage | Crack1 Width(mm) | Crack2 Width(mm) | Crack3 Width(mm) |
| --- | --- | --- | --- | --- |
| 2013/10/17 | 8 | 1 | 1 | 0 |
| 2013/10/19 | 21 | 2 | 4 | 6 |
| 2013/10/20 | 33 | 9 | 11 | 11 |
| 2013/10/21 | 44 | 15 | 17 | 16 |
| 2013/10/23 | 62 | 11 | 11 | 11 |
| 2013/10/24 | 83.3 | 0 | 1 | 2 |
| 2013/10/25 | 140 | 4 | 6 | 5 |
| 2013/10/26 | 150 | 7 | 7 | 6 |
| 2013/10/28 | 158 | 5 | 4 | 3 |
| 2013/10/30 | 177 | 3 | 2 | 1 |
| 2013/11/2 | 187 | 0 | 1 | 0 |
| 2013/11/4 | 213 | 0 | 1 | 0 |

Supplementary data for Figure 3a1: Surface soil water loss in the BLT site

| Distance | | Day1 | | Day2 | | Day3 | | Day4 | | Day5 | | Day6 | |
| --- | --- | --- | --- | --- | --- | --- | --- | --- | --- | --- | --- | --- | --- |
| 10 | | 0.1493 | | 0.339 | | 0.5882 | | 0.6452 | | 0.7563 | | 0.6667 | |
| 20 | | 0.0746 | | 0.2825 | | 0.4202 | | 0.4032 | | 0.5042 | | 0.6 | |
| 30 | | 0.0746 | | 0 | | 0.2521 | | 0.1613 | | 0.3361 | | 0.4667 | |
| 50 | | 0.1493 | | 0.0565 | | 0 | | 0.1613 | | 0.084 | | 0.2 | |
| 75 | | 0 | | 0 | | -0.084 | | 0.1613 | | 0 | | 0.0667 | |
| 100 | | 0.0746 | | 0 | | -0.084 | | 0 | | -0.084 | | -0.667 | |
| Day7 | Day8 | | Day9 | | Day10 | | Day11 | | Day12 | | Day13 | |  |
| 1.2195 | 0.9848 | | 0.5202 | | 0.5333 | | 0.6 | | 0.9524 | | 0.6618 | |  |
| 1.0569 | 0.6818 | | 0.4624 | | 0.3333 | | 0.5333 | | 0.7143 | | 0.5147 | |  |
| 0.813 | 0.3788 | | 0.4046 | | 0.2667 | | 0.4 | | 0.5556 | | 0.4412 | |  |
| 0.3 | 0.2273 | | 0.1156 | | 0.1333 | | 0.0667 | | 0.2381 | | 0.2941 | |  |
| 0 | 0.1515 | | 0 | | 0.0667 | | 0.2 | | 0.0794 | | 0.0735 | |  |
| 0 | 0 | | -0.0578 | | 0 | | 0.667 | | 0 | | 0.1471 | |  |
| Day14 | Day15 | | Day16 | | Day17 | | Day18 | | Day19 | |  |  |  |
| 0.614 | 0.4959 | | 0.2459 | | 0 | | 0 | | -0.1 | |  |  |  |
| 0.5263 | 0.3306 | | 0.1639 | | 0.0794 | | 0.1 | | 0 | |  |  |  |
| 0.3509 | 0.1653 | | 0.1639 | | -0.0794 | | 0 | | 0 | |  |  |  |
| 0.2632 | 0.0826 | | 0.2459 | | 0.1587 | | -0.1 | | 0.1 | |  |  |  |
| 0.2632 | 0 | | 0.082 | | 0.0794 | | 0.1 | | 0 | |  |  |  |
| 0.0877 | -0.1653 | | 0.082 | | 0 | | 0 | | -0.1 | |  |  |  |

Supplementary data for Figure 3a2: Surface soil water loss in the BDLT site

| Distance | | Oct.30/Water loss (%) | Nov.2/Water loss (%) | | Nov.3/Water loss (%) | Nov.5/Water loss (%) |  |
| --- | --- | --- | --- | --- | --- | --- | --- |
| 10cm | | 1 | 2.2 | | 2 | 2.4 |  |
| 20cm | | 0.9 | 2 | | 1.9 | 2.1 |  |
| 30cm | | 0.7 | 1.9 | | 1.4 | 1.9 |  |
| 40cm | | 0.4 | 1.6 | | 1.1 | 1.8 |  |
| 50cm | | 0.2 | 1.2 | | 1.233333 | 1.533333 |  |
| 60cm | | 0.1 | 1.3 | | 0.533333 | 1 |  |
| 70cm | | -0.06667 | 0.9 | | 0.633333 | 0.6 |  |
| 80cm | | 0.133333 | 0.466667 | | 0.233333 | 0.4 |  |
| 90cm | | -0.1 | 0.3 | | 0.233333 | 0.1 |  |
| 100cm | | 0 | 0.1 | | 0.1 | -0.1 |  |
| Nov.7/Water loss (%) | Nov.9/Water loss (%) | | Nov.13/Water loss (%) | Nov.15/Water loss (%) | | Nov.17/Water loss (%) | |
| 0.733333 | 1.7 | | 1.2 | 0.233333 | | 0.3 | |
| 0.833333 | 1.166667 | | 1.1 | 0.333333 | | 0.3 | |
| 0.9 | 0.7 | | 1 | 0.533333 | | 0.1 | |
| 1.2 | 0.6 | | 0.7 | 0.333333 | | 0.1 | |
| 0.266667 | 0.3 | | 0.4 | -0.06667 | | 0.4 | |
| 0.166667 | 0.1 | | 0.3 | -0.06667 | | 0.366667 | |
| -0.06667 | -0.03333 | | -0.1 | 0.233333 | | -0.26667 | |
| -0.2 | 0.233333 | | 0 | 0.033333 | | 0.1 | |
| -0.36667 | 0.266667 | | 0 | 0 | | -0.1 | |
| -0.06667 | -0.1 | | -0.2 | 0.1 | | 0.066667 | |

Supplementary data for Figure3d1: Water content in the Sandstone in the DLT site

| Depth（m） | Water content at unmined stage（%） | Water content at place between unmined and mining stages（%） | Water content at mining stage（%） | Water content at post-mining stage（%） |
| --- | --- | --- | --- | --- |
| 0 | 5.74 | 9.37 | 6.53 | 6.45 |
| 0.5 | 4.8 | 4.87 | 5.36 | 5.17 |
| 1 | 4.56 | 4.92 | 5.34 | 5.12 |
| 1.5 | 4.23 | 4.63 | 4.23 | 5.5 |
| 2 | 5.96 | 4.98 | 4.12 | 7.54 |
| 2.5 | 8.4 | 9.44 | 3.12 | 4.97 |
| 3 | 7.22 | 5.79 | 3.21 | 5.16 |
| 3.5 | 7.99 | 6.1 | 6.53 | 5.33 |
| 4 | 8.27 | 6 | 5.36 | 4.49 |
| 4.5 | 9.54 | 4.52 | 5.49 | 6.1 |
| 5 | 3.84 | 7.68 | 5.62 | 8.36 |
| 5.5 | 2.84 | 13.04 | 5.75 | 6.95 |
| 6 | 3.15 | 3.65 | 5.88 | 6.23 |
| 6.5 | 3.86 | 7.71 | 6.01 | 5.51 |
| 7 | 4.42 | 6.02 | 3.41 | 5.23 |
| 7.5 | 8.01 | 4.23 | 3.34 | 4.07 |
| 8 | 3.43 | 3.48 | 3.26 | 4.56 |
| 8.5 | 4.17 | 5.92 | 3.19 | 2.63 |
| Average value | 5.58 | 6.24 | 4.76 | 5.52 |

Supplementary data for Figure3d2: Water content in the Clay in the DLT site

| Depth（m） | Water content at unmined stage（%） | Water content at place between unmined and mining stages（%） | Water content at mining stage（%） | Water content at post-mining stage（%） |
| --- | --- | --- | --- | --- |
| 0 | 18.34 | 21.36 | 15.23 | 25.97 |
| 0.5 | 19.37 | 20.85 | 15.45 | 25.9 |
| 1 | 20.04 | 22.87 | 15.76 | 29.96 |
| 1.5 | 23.93 | 23.3 | 19.04 | 28.45 |
| 2 | 23.69 | 23.9 | 29.12 | 31.89 |
| 2.5 | 26.43 | 31.48 | 21.64 | 21.86 |
| 3 | 24.37 | 23.11 | 25.55 | 19.65 |
| 3.5 | 22.77 | 22.41 | 24.25 | 17.43 |
| 4 | 20.97 | 21.12 | 23.35 | 20.21 |
| 4.5 | 25.55 | 21.82 | 21.65 | 22.99 |
| 5 | 26.42 | 20.35 | 20.35 | 25.77 |
| 5.5 | 24.87 | 21.16 | 19.05 | 23.7 |
| 6 | 13.7 | 20.73 | 21.54 | 18.61 |
| 6.5 | 16.4 | 19.51 | 19.23 | 12.15 |
| 7 | 20.52 | 24.34 | 18.25 | 11.22 |
| 7.5 | 20.27 | 15.62 | 19.56 | 11.68 |
| 8 | 20.03 | 16.19 | 16.23 | 9.83 |
| 8.5 | 19.78 | 24.21 | 17.1 | 10.29 |
| 9 | 19.54 | 20.71 | 15.56 | 10.76 |
| Average value | 21.42 | 21.84 | 19.89 | 19.91 |

Supplementary data for Figure4: the soil physical and chemical indicators in the DLT site

| Bulk Desity  (g/cm^3) | WF B^’^ | 1.598 | 0.0132 | 1.6386 | 0.0031 | 1.6685 | 0.00805 |
| --- | --- | --- | --- | --- | --- | --- | --- |
| Bulk Desity  (g/cm^3) | WF A^’^ | 1.534 | 0.0115 | 1.5935 | 0.00847 | 1.65049 | 0.00747 |
|  |  |  |  |  |  |  |  |
| Porosity(%) | WF B^’^ | 39.69 | 0.4986 | 38.329 | 0.20045 | 37.0376 | 0.3036 |
| Porosity(%) | WF A^’^ | 42.11 | 0.2646 | 39.869 | 0.33637 | 37.7173 | 0.41676 |
|  |  |  |  |  |  |  |  |
| Moisiture Content(%) | WF B^’^ | 2.495 | 0.1309 | 2.9477 | 0.03619 | 3.70423 | 0.30604 |
| Moisiture Content(%) | WF A^’^ | 2.964 | 0.1007 | 3.5684 | 0.06108 | 5.38415 | 0.5328 |
|  |  |  |  |  |  |  |  |
| pH Value | WF B^’^ | 7.99 | 0.125 | 8 | 0.251 | 7.98 | 0.325 |
| pH Value | WF A^’^ | 7.959 | 0.09 | 7.9557 | 0.054 | 7.95143 | 0.048 |
|  |  |  |  |  |  |  |  |
| Phosphorus  (mg/kg) | WF B^’^ | 1.382 | 0.0121 | 1.5325 | 0.00652 | 1.77632 | 0.01119 |
| Phosphorus  (mg/kg) | WF A^’^ | 1.822 | 0.0038 | 2.1215 | 0.01342 | 2.44889 | 0.0064 |
|  |  |  |  |  |  |  |  |
| Potassium  (mg/kg) | WF B^’^ | 15.2 | 0.6633 | 17.6 | 0.5099 | 24 | 0.54772 |
| Potassium  (mg/kg) | WF A^’^ | 11 | 2 | 13.4 | 1.50333 | 18.4 | 1.2083 |
|  |  |  |  |  |  |  |  |
| Organic Matter  (mg/kg)x10^3 | WF B^’^ | 3.254 | 0.6737 | 3.1426 | 0.40345 | 3.74308 | 0.41988 |
| Organic Matter  (mg/kg)x10^3 | WF A^’^ | 2.568 | 0.4292 | 2.7805 | 0.19928 | 3.46228 | 0.47298 |
|  |  |  |  |  |  |  |  |
| Total Nigroger  (mg/kg) | WF B^’^ | 0.017 | 0.0013 | 0.022 | 0.0021 | 0.0264 | 4.00E-04 |
| Total Nigroger  (g/kg) | WF A^’^ | 0.026 | 0.0039 | 0.0233 | 0.00271 | 0.02843 | 0.00364 |

All other data of this study are available from the corresponding author upon reasonable request, i.e. AutoCAD data for Figure 2a, electrical prospecting data for Figure 3b and seismic data for figure 2c and 3c.
